# Knowledge for effective action to improve the health of women, children and adolescents in the post-2015 era: a call for papers

**DOI:** 10.2471/BLT.15.156521

**Published:** 2015-05-01

**Authors:** Flavia Bustreo, Robin Gorna

**Affiliations:** a World Health Organization, avenue Appia 20, 1211 Geneva 27, Switzerland.; b Partnership for Maternal, Newborn & Child Health, Geneva, Switzerland.

In 2010, the United Nations Secretary-General launched the *Global strategy for women’s and children’s health* for 2010–2015,^1^ intended to accelerate progress towards the Millennium Development Goals (MDGs) 4, 5 and 6. There has been significant progress, but more is needed, especially for women’s and children’s health. Since 1990, child mortality fell by 49% and maternal mortality by 45%. These are substantial improvements, but still insufficient to meet the targets of 67% and 75% reductions in child and maternal mortality respectively.^2^^,^^3^ Over 200 million women have unmet needs for family planning – far short of the agreed MDG target of universal access to reproductive health care.^4^

The Global Strategy has catalysed progress:^5^ the health of women and children is high on the political agenda. Over 300 stakeholders – including governments, multilateral organizations, nongovernmental organizations, donors, the private sector, academic institutes and health-care professional associations – recently made 400 commitments, raising over US$ 45 billion new financing. New global initiatives were launched for priority issues, including family planning, newborn and child survival; over 1000 innovations have been selected and supported, and a shared accountability framework was a landmark for women’s and children’s health. 

An intensified focus on country implementation is now needed, in the final phase of the MDGs and the inception of the new Sustainable Development Goals. Evidence exists to support country implementation – on essential health interventions, strengthening the health workforce and systems, and enabling policies for health. A global investment framework for women’s and children’s health specifies the high-impact investments required and shows that these investments not only save lives, but can also yield nine times the investment value in social and economic benefits.^6^

The post-2015 health and development agenda highlights new challenges and opportunities. Access to sexual and reproductive health information, goods and services is essential for adults and adolescents to plan families, avoid unsafe abortions and prevent infections, including human immunodeficiency virus (HIV) and human papillomavirus (HPV). Significant inequalities exist in access to health care within and between countries.^7^ Uneven provision of reproductive, maternal, newborn, child and adolescent health services, and siloed programmes such as for immunization, malaria, tuberculosis and HIV, remain a challenge in some countries with a need to integrate and link programmes to meet the holistic health needs of individuals and populations. Evidence is growing on the potential of human rights-based approaches to address inequalities and improve health, however, more evidence is required on the most effective methods of implementation and assessment of impact.^8^ Over half the preventable deaths in mothers, newborns and children under the age of five occur in settings of conflict, displacement or natural disasters.^9^ We must consider how to make essential health services more resilient to shifts in political and environmental contexts – from normalcy, to fragility, to crisis and back. 

The substantial reductions in maternal and child mortality since 1990 resulted, in large part, from better education, gender equality, improved water, sanitation and hygiene, infrastructure and technology, poverty reduction and good governance.^10^ We need to understand how countries can promote policies to address the interrelated determinants of health and sustainable development through the core work of different sectors, and when this needs to be supplemented by intersectoral collaboration. 

National leadership is crucial and spans political commitment; ensuring that there is robust, relevant and timely evidence to inform policy-making; advocacy and resource mobilization; systems strengthening; alignment of stakeholders and partnerships; intersectoral coordination; improving access to essential quality care; scaling-up of innovations; human rights and equity-based approaches. Country implementation and investment plans, resources and results need to drive action and form the basis of accountability of all stakeholders.

More knowledge is required from analyses of lessons from the MDG process and to ascertain effective implementation approaches to meet the post-2015 challenges. The *Global strategy for women’s, children’s and adolescents’ health*, intended to accelerate progress on these issues, will be launched in September this year.^11^

For the global community to accelerate progress towards improving women’s, children’s and adolescents’ health and achieving the Sustainable Development Goals, we need knowledge to be translated into stronger action. To this end, we are now calling for submissions to a theme issue of the *Bulletin* on the health of women, children and adolescents informed by lessons learned from the MDGs and evidence on effective country implementation. The deadline for submissions is 31 December 2015. Manuscripts should follow the *Bulletin’s* guidelines for contributors, specify this call for papers in the covering letter and be submitted via http://submit.bwho.org.

## References

[1] Global strategy for women's and children's health. New York: Partnership for Maternal, Newborn and Child Health; 2010. Available from: http://www.who.int/pmnch/activities/advocacy/fulldocument_globalstrategy [cited 2015 April 14].

[2] Levels and Trends in Child Mortality. Report 2014. Estimates Developed by the UN Inter-agency Group for Child Mortality Estimation. New York: UNICEF, WHO, The World Bank and the United Nations Population Programme; 2014. Available from: http://www.childmortality.org/files_v19/download/unicef-2013-child-mortality-report-LR-10_31_14_195.pdf[cited 2015 April 14].

[3] Trends in Maternal Mortality: 1990 to 2013. Estimates by WHO, UNICEF, UNFPA, The World Bank and the United Nations Population Division. Geneva: World Health Organization: 2014. Available from: http://apps.who.int/iris/bitstream/10665/112682/2/9789241507226_eng.pdf[cited 2015 April 14].

[4] Women and health: 20 years of the Beijing Declaration and Platform for Action. Report by the Secretariat. Executive Board 136 Session, Provisional agenda item 7.4, EB136/18, 24 December 2014. Geneva: World Health Organization: 2014. Available from: http://apps.who.int/gb/ebwha/pdf_files/EB136/B136_18-en.pdf [cited 2015 April 14].

[5] Saving Lives, Protecting Futures. Progress Report on the Global Strategy for Women’s and Children’s Health 2010-2015. Washington DC: Every Woman, Every Child, UN Foundation: 2015. Available from: http://everywomaneverychild.org/images/EWEC_Progress_Report_FINAL_3.pdf

[6] Stenberg K, Axelson H, Sheehan P, Anderson I, Gülmezoglu AM, Temmerman M, et al.; Study Group for the Global Investment Framework for Women’s Children’s Health. Advancing social and economic development by investing in women’s and children’s health: a new Global Investment Framework. Lancet. 2014 4 12;383(9925):1333–54. 10.1016/S0140-6736(13)62231-X 24263249

[7] Countdown to 2015 and beyond: fulfilling the health agenda for women and children. Geneva: World Health Organization & UNICEF; 2014. Available from: http://www.countdown2015mnch.org/reports-and-articles/2014-report [cited 2015 April 14].

[8] Bustreo F, Hunt P, Gruskin S, Eide A, McGoey L, Rao S, et al. Women’s and children’s health: Evidence of impact of human rights. Geneva: World Health Organization; 2013. Available from: http://www.who.int/maternal_child_adolescent/documents/women_children_human_rights/en/ [cited 2015 April 14].

[9] Fragile states 2014: domestic revenue mobilisation in fragile states [statistics from references 2 and 3 on interagency mortality estimates and OECD]. Paris: Organisation for Economic Co-operation and Development; 2014. Available from: http://www.oecd.org/dac/governance-peace/conflictandfragility/docs/FSR-2014.pdf [cited 2015 April 14].

[10] Kuruvilla S, Schweitzer J, Bishai D, Chowdhury S, Caramani D, Frost L, et al.; Success Factors for Women’s and Children’s Health study groups. Success factors for reducing maternal and child mortality. Bull World Health Organ. 2014 7 1;92(7):533–44B. 10.2471/BLT.14.138131 25110379 PMC4121875

[11] Every Woman Every Child [website]. Shaping the future for healthy women, children and adolescents: learn more about the process to update the global strategy. New York: United Nations Foundation; 2015. Available from: Available from: http://www.everywomaneverychild.org/global-strategy-2

